# Analysis of the Sonic Hedgehog Signaling Pathway in Normal and Abnormal Bladder Development

**DOI:** 10.1371/journal.pone.0053675

**Published:** 2013-01-07

**Authors:** Kristin R. DeSouza, Monalee Saha, Ashley R. Carpenter, Melissa Scott, Kirk M. McHugh

**Affiliations:** Center for Molecular and Human Genetics, The Research Institute at Nationwide Children's Hospital, Columbus, Ohio, United States of America; National Cancer Institute, United States of America

## Abstract

In this study, we examined the expression of Sonic Hedgehog, Patched, Gli1, Gli2, Gli3 and Myocardin in the developing bladders of male and female normal and megabladder (*mgb−/−*) mutant mice at embryonic days 12 through 16 by *in situ* hybridization. This analysis indicated that each member of the Sonic Hedgehog signaling pathway as well as Myocardin displayed distinct temporal and spatial patterns of expression during normal bladder development. In contrast, *mgb−/−* bladders showed both temporal and spatial changes in the expression of Patched, Gli1 and Gli3 as well as a complete lack of Myocardin expression. These changes occurred primarily in the outer mesenchyme of developing *mgb−/−* bladders consistent with the development of an amuscular bladder phenotype in these animals. These results provide the first comprehensive analysis of the Sonic Hedgehog signaling pathway during normal bladder development and provide strong evidence that this key signaling cascade is critical in establishing radial patterning in the developing bladder. In addition, the lack of detrusor smooth muscle development observed in *mgb−/−* mice is associated with bladder-specific temporospatial changes in Sonic Hedgehog signaling coupled with a lack of Myocardin expression that appears to result in altered patterning of the outer mesenchyme and poor initiation and differentiation of smooth muscle cells within this region of the developing bladder.

## Introduction

**Figure 1:**
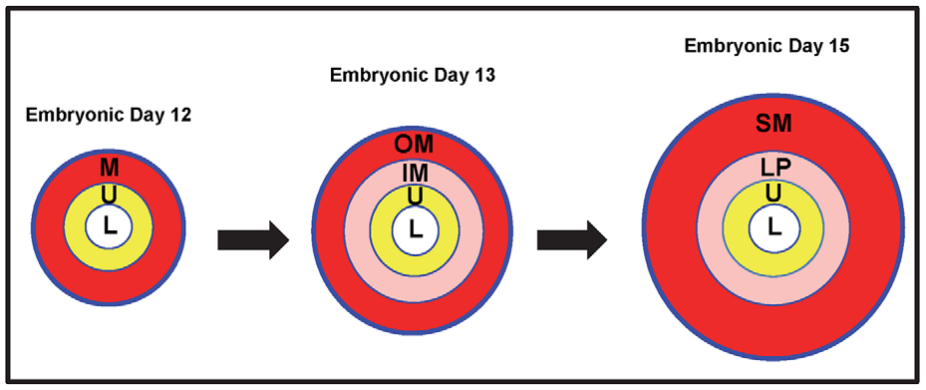
Model of murine bladder development. Timeline showing the approximate embryonic stage at which the lumen (L), urothelium (U), inner mesenchyme (IM), outer mesenchyme (OM), lamina propria (LP) and detrusor smooth muscle (SM) first become evident.

The mammalian urinary bladder develops from a partitioning of the cloaca by the urorectal septum with the superior region of the developing urogenital sinus, giving rise to the definitive urinary bladder [1], [2]. The developing bladder is initially composed of three distinct layers that include the 1) innermost urothelium, 2) intermediate-placed mesenchyme and 3) outer serosal layer (Fig. 1) [2], [3]. The innermost urothelium is a derivative of the embryonic endoderm and is composed of an expandable transitional epithelium that provides a watertight barrier for urine storage [4], [5]. The mesodermal-derived bladder mesenchyme initially differentiates into two distinct compartments that include the inner and outer mesenchyme. The inner mesenchyme, adjacent to the urothelium, differentiates into the lamina propria composed of loose connective tissue [1], [4], [5]. This layer enables the bladder to expand as it fills, while still maintaining a low pressure to protect the kidneys. The outer mesenchyme differentiates into detrusor smooth muscle that generates the tensile strength and functional contractility necessary to store and expel urine [1], [4], [5].

Bladder organogenesis is highly dependent upon the reciprocal interactions of differentiating urothelium and bladder mesenchyme [6]–[8]. Tissue recombination experiments indicate that bladder urothelium is required to induce bladder mesenchyme to differentiate into smooth muscle via a diffusible signaling molecule [3], [7], [9]. Of particular interest, Sonic Hedgehog (*Shh*) and it's downstream signaling molecules have been shown to be involved in many epithelial-mesenchymal interactions during development, including proper bladder development and differentiation [10]–[16]. *Shh* is a secreted signaling molecule that interacts with the transmembrane receptor Patched (*Ptch*) resulting in repression of *Ptch*, and activation of *Gli* transcription factors in the target cell. Work by numerous labs indicates that this canonical Shh signaling pathway is involved in multiple processes, including cell fate determination, morphogenesis, differentiation, and apoptosis [13], [17].

Our lab has identified a unique transgenic mouse model for studying bladder development, function, and pathogenesis [18]. Homozygous megabladder (*mgb−/−*) mice develop greatly enlarged bladders *in utero* as a result of a tissue specific defect in detrusor smooth muscle development. In this current study, we characterized the expression patterns of the *Shh* ligand, *Ptch* receptor, downstream transcription factors *Gli1, Gli2*, & *Gli3* and a potential target gene of the pathway, Myocardin (*Myocd*), in the bladders of male and female wild type (normal) and *mgb−/−* (mutant) mice at key developmental time-points for smooth muscle differentiation-embryonic days (E) 12, 13, 14, 15, and 16- by *in situ* hybridization. This analysis indicated that members of the *Shh* signaling pathway and *Myocd* were expressed in distinct temporal and spatial patterns during normal bladder development. The observed patterns of expression were consistent with the Shh signaling pathway playing a key role in radial patterning of the developing bladder. Defects in the expression of *Ptch*, *Gli1* and *Gli3* in developing *mgb−/−* bladders suggest that changes in radial patterning coupled with a lack of *Myocd* expression are responsible for the lack of detrusor smooth muscle development in these animals.

## Results

**Figure 2:**
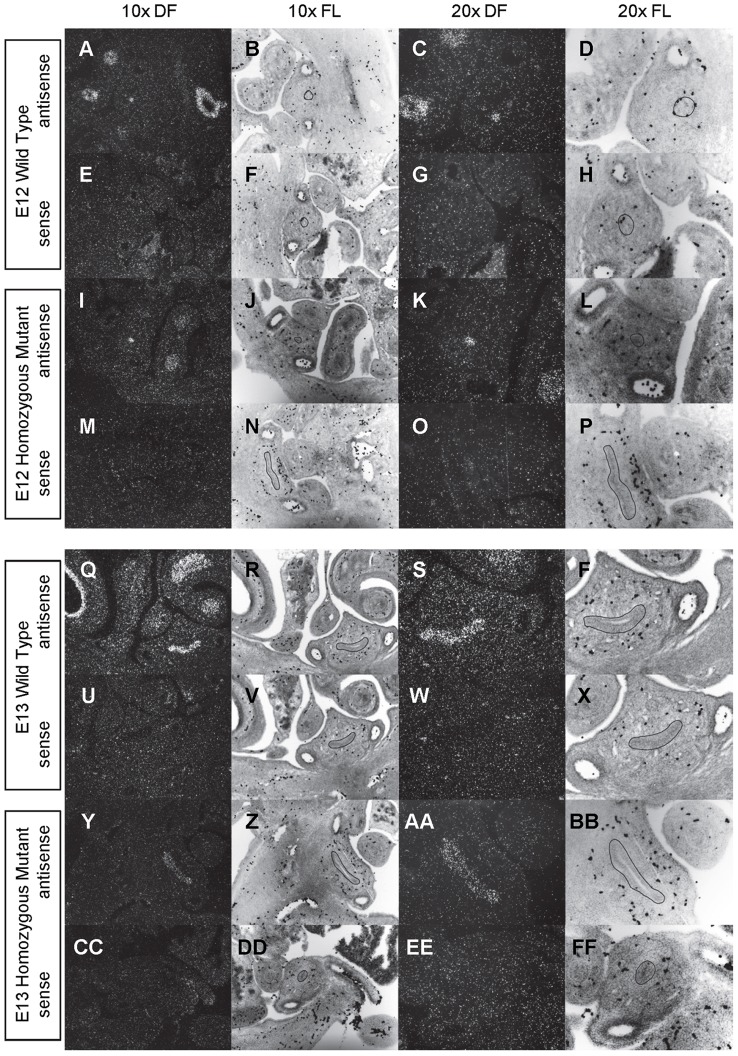
Expression of *Shh* in the bladder of E12 and E13 mice. *In situ* hybridization analysis of *Shh* expression in E12 (A-P) and E13 (Q-FF) bladders using antisense (A-D, I-L, Q-T, Y-BB) and sense (E-H, M-P, U-X, CC-FF) riboprobes on transverse sections of wild type (A-H, Q-X) and *mgb−/−* (I-P, Y-FF) mice. Sections are shown at 10X dark field (DF; A, E, I, M, Q, U, Y, CC), 10X fluorescence (FL; B, F, J, N, R, V, Z, DD), 20X dark field (DF; C, G, K, O, S, W, AA, EE) and 20X fluorescence (FL; D, H, L, P, T, X, BB, FF). The basement membrane of the developing urothelium has been outlined in black as a point of reference in 10X and 20X FL views.

**Figure 3:**
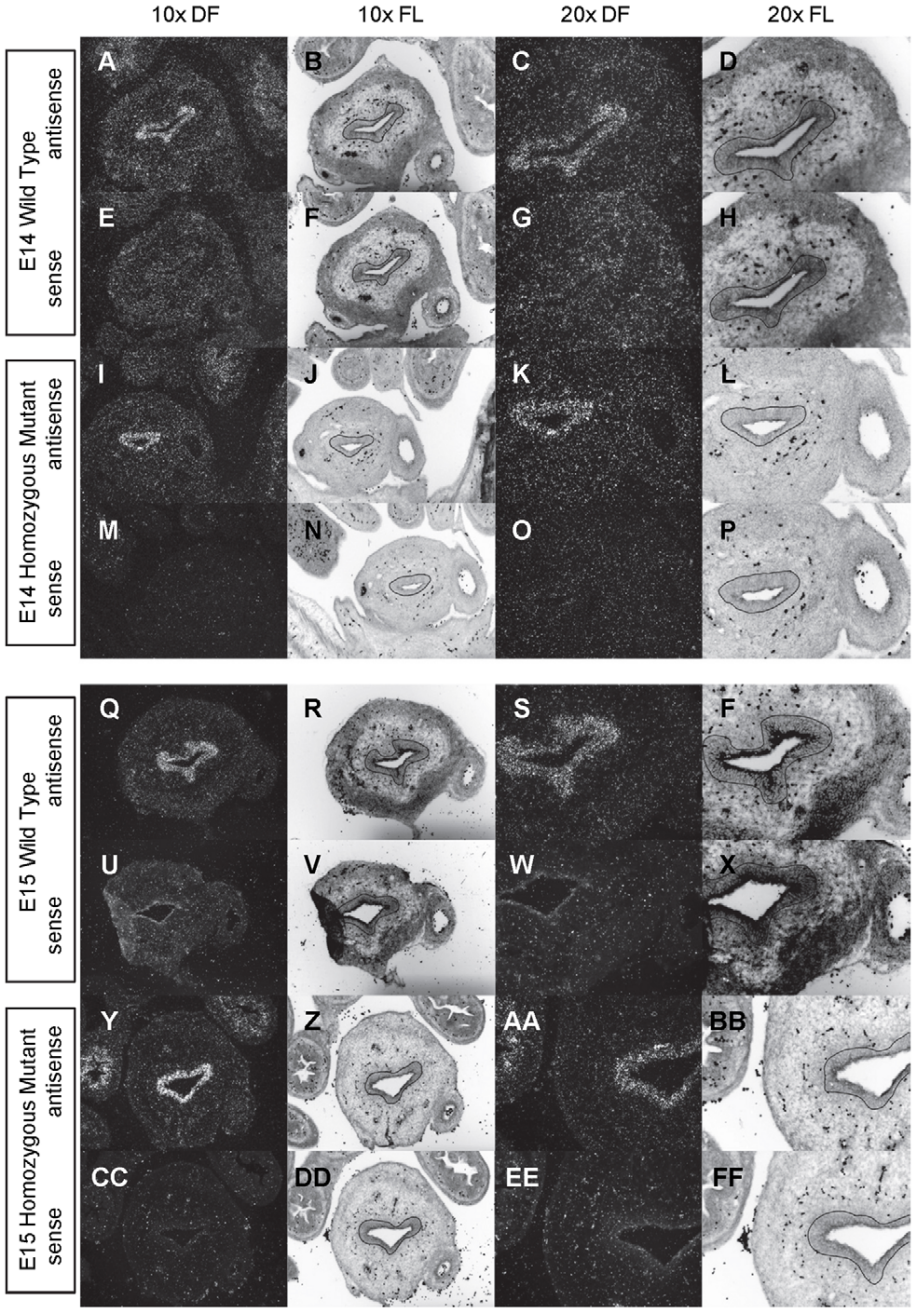
Expression of *Shh* in the bladder of E14 and E15 mice. *In situ* hybridization analysis of *Shh* expression in E14 (A-P) and E15 (Q-FF) bladders using antisense (A-D, I-L, Q-T, Y-BB) and sense (E-H, M-P, U-X, CC-FF) riboprobes on transverse sections of wild type (A-H, Q-X) and *mgb−/−* (I-P, Y-FF) mice. Sections are shown at 10X dark field (DF; A, E, I, M, Q, U, Y, CC), 10X fluorescence (FL; B, F, J, N, R, V, Z, DD), 20X dark field (DF; C, G, K, O, S, W, AA, EE) and 20X fluorescence (FL; D, H, L, P, T, X, BB, FF). The basement membrane of the developing urothelium has been outlined in black as a point of reference in 10X and 20X FL views.

**Figure 4:**
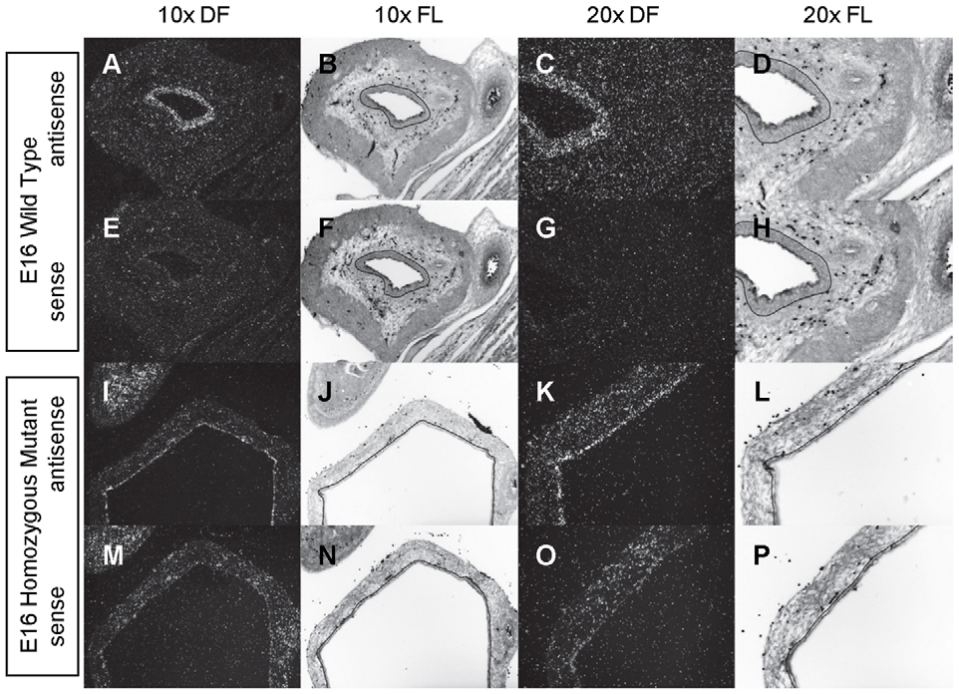
Expression of *Shh* in the bladder of E16 mice. *In situ* hybridization analysis of *Shh* expression in E16 (A-P) bladders using antisense (A-D, I-L) and sense (E-H, M-P) riboprobes on transverse sections of wild type (A-H) and *mgb−/−* (I-P) mice. Sections are shown at 10X dark field (DF; A, E, I, M), 10X fluorescence (FL; B, F, J, N), 20X dark field (DF; C, G, K, O) and 20X fluorescence (FL; D, H, L, P). The basement membrane of the developing urothelium has been outlined in black as a point of reference in 10X and 20X FL views.

**Figure 5:**
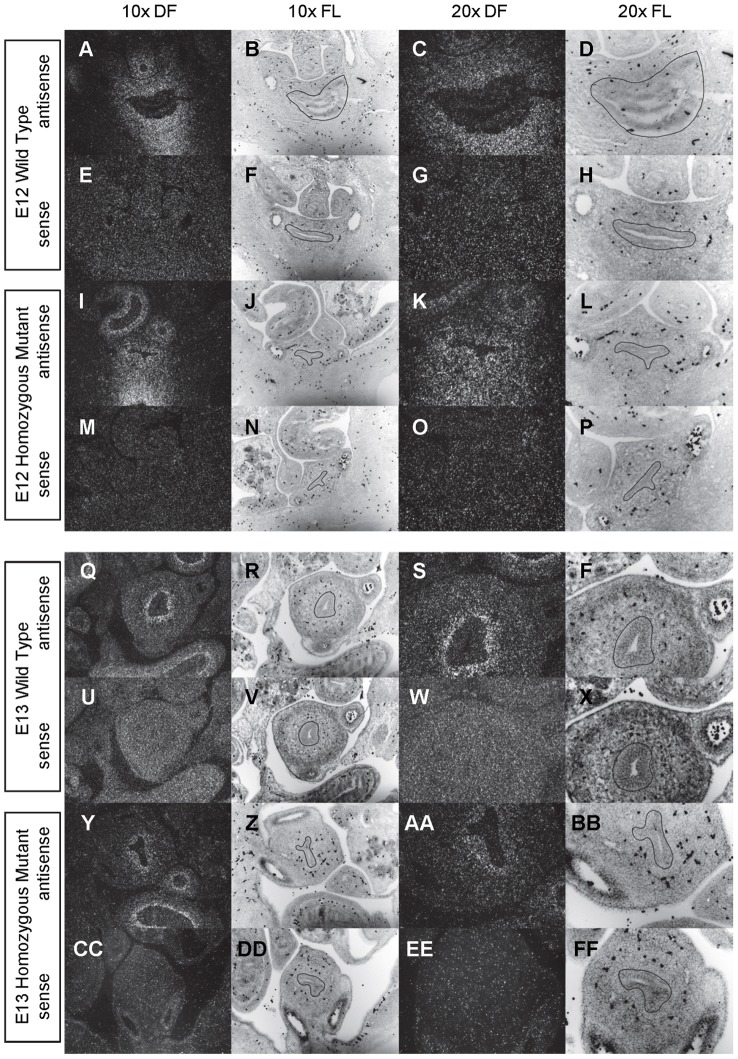
Expression of *Ptch* in the bladder of E12 and E13 mice. *In situ* hybridization analysis of *Ptch* expression in E12 (A-P) and E13 (Q-FF) bladders using antisense (A-D, I-L, Q-T, Y-BB) and sense (E-H, M-P, U-X, CC-FF) riboprobes on transverse sections of wild type (A-H, Q-X) and *mgb−/−* (I-P, Y-FF) mice. Sections are shown at 10X dark field (DF; A, E, I, M, Q, U, Y, CC), 10X fluorescence (FL; B, F, J, N, R, V, Z, DD), 20X dark field (DF; C, G, K, O, S, W, AA, EE) and 20X fluorescence (FL; D, H, L, P, T, X, BB, FF). The basement membrane of the developing urothelium has been outlined in black as a point of reference in 10X and 20X FL views.

**Figure 6:**
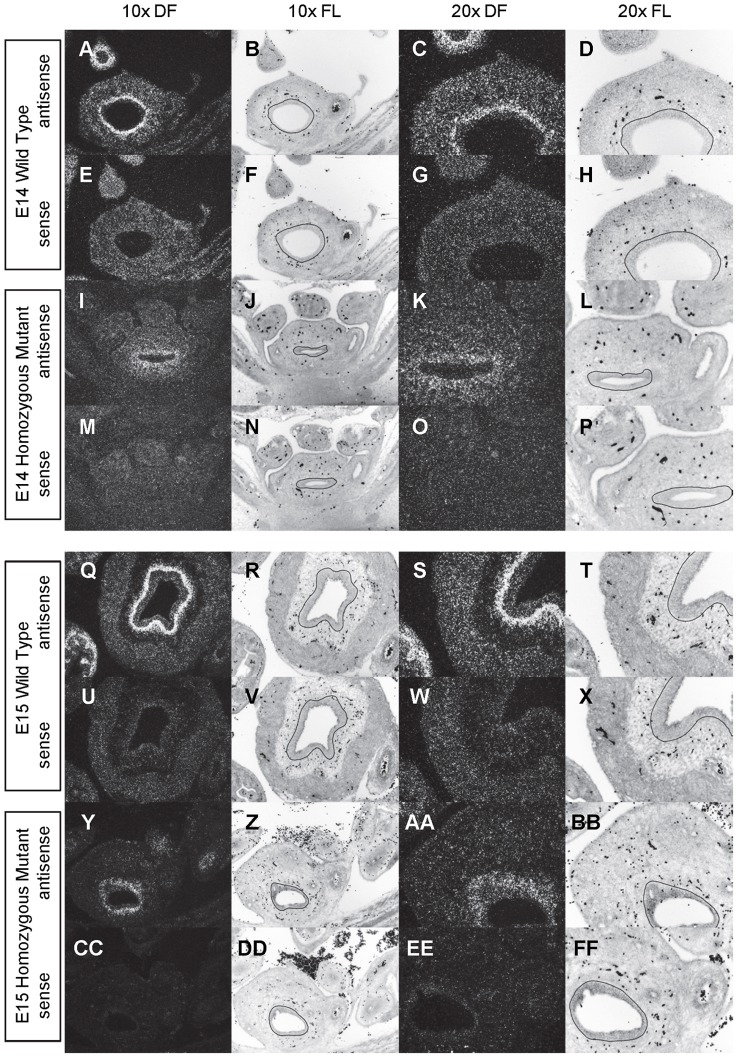
Expression of *Ptch* in the bladder of E14 and E15 mice. *In situ* hybridization analysis of *Ptch* expression in E14 (A-P) and E15 (Q-FF) bladders using antisense (A-D, I-L, Q-T, Y-BB) and sense (E-H, M-P, U-X, CC-FF) riboprobes on transverse sections of wild type (A-H, Q-X) and *mgb−/−* (I-P, Y-FF) mice. Sections are shown at 10X dark field (DF; A, E, I, M, Q, U, Y, CC), 10X fluorescence (FL; B, F, J, N, R, V, Z, DD), 20X fluorescence (DF; C, G, K, O, S, W, AA, EE) and 20X fluorescence (FL; D, H, L, P, T, X, BB, FF). The basement membrane of the developing urothelium has been outlined in black as a point of reference in 10X and 20X FL views.

**Figure 7:**
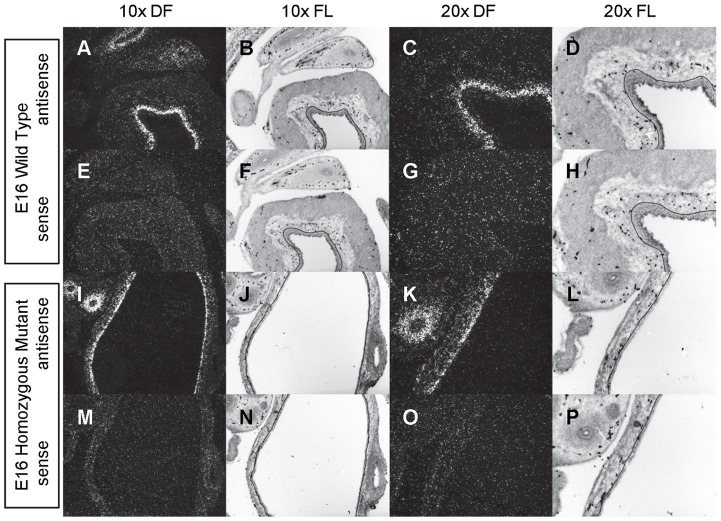
Expression of *Ptch* in the bladder of E16 mice. *In situ* hybridization analysis of *Ptch* expression in E16 (A-P) bladders using antisense (A-D, I-L) and sense (E-H, M-P) riboprobes on transverse sections of wild type (A-H) and *mgb−/−* (I-P) mice. Sections are shown at 10X dark field (DF; A, E, I, M), 10X fluorescence (FL; B, F, J, N), 20X fluorescence (DF; C, G, K, O) and 20X (FL; D, H, L, P). The basement membrane of the developing urothelium has been outlined in black as a point of reference in 10X and 20X FL views.

**Figure 8:**
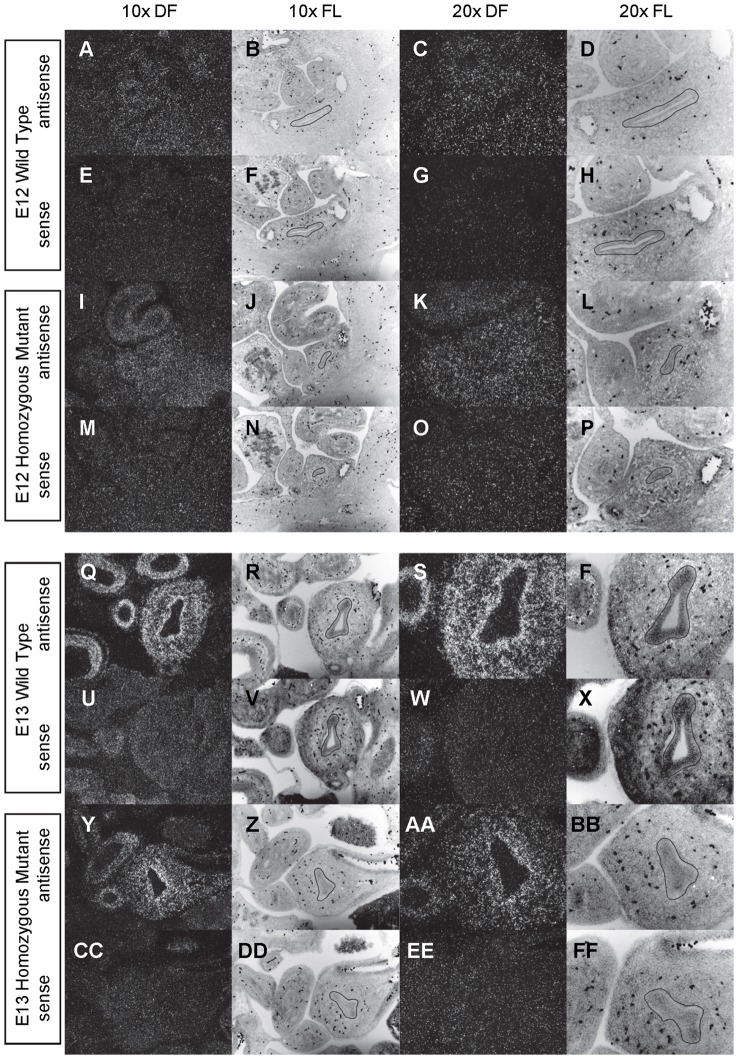
Expression of *Gli1* in the bladder of E12 and E13 mice. *In situ* hybridization analysis of *Gli1* expression in E12 (A-P) and E13 (Q-FF) bladders using antisense (A-D, I-L, Q-T, Y-BB) and sense (E-H, M-P, U-X, CC-FF) riboprobes on transverse sections of wild type (A-H, Q-X) and *mgb−/−* (I-P, Y-FF) mice. Sections are shown at 10X dark field (DF; A, E, I, M, Q, U, Y, CC), 10X fluorescence (FL; B, F, J, N, R, V, Z, DD), 20X dark field (DF; C, G, K, O, S, W, AA, EE) and 20X fluorescence (FL; D, H, L, P, T, X, BB, FF). The basement membrane of the developing urothelium has been outlined in black as a point of reference in 10X and 20X FL views.

**Figure 9:**
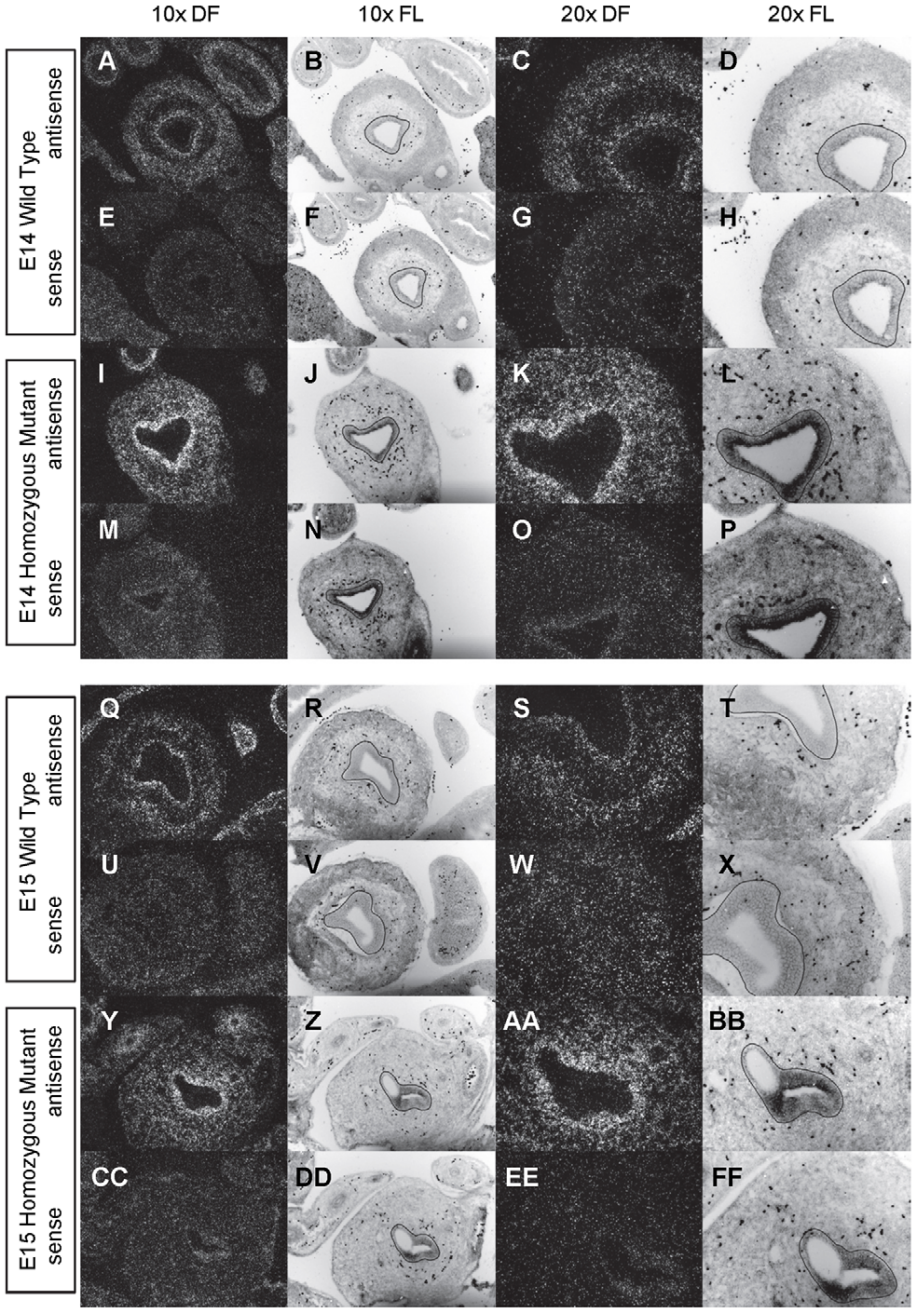
Expression of *Gli1* in the bladder of E14 and E15 mice. *In situ* hybridization analysis of *Gli1* expression in E14 (A-P) and E15 (Q-FF) bladders using antisense (A-D, I-L, Q-T, Y-BB) and sense (E-H, M-P, U-X, CC-FF) riboprobes on transverse sections of wild type (A-H, Q-X) and *mgb−/−* (I-P, Y-FF) mice. Sections are shown at 10X dark field (DF; A, E, I, M, Q, U, Y, CC), 10X fluorescence (FL; B, F, J, N, R, V, Z, DD), 20X dark field (DF; C, G, K, O, S, W, AA, EE) and 20X fluorescence (FL; D, H, L, P, T, X, BB, FF). The basement membrane of the developing urothelium has been outlined in black as a point of reference in 10X and 20X FL views.

**Figure 10:**
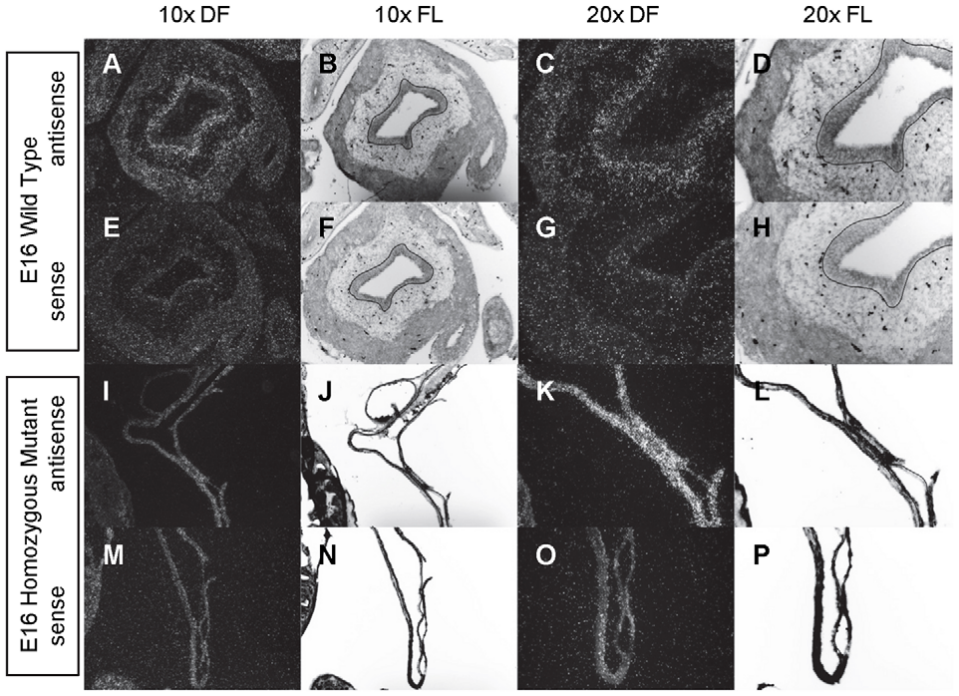
Expression of *Gli1* in the bladder of E16 mice. *In situ* hybridization analysis of *Gli1* expression in E16 (A-P) bladders using antisense (A-D, I-L) and sense (E-H, M-P) riboprobes on transverse sections of wild type (A-H) and *mgb−/−* (I-P) mice. Sections are shown at 10X dark field (DF; A, E, I, M), 10X fluorescence (FL; B, F, J, N), 20X dark field (DF; C, G, K, O) and 20X fluorescence (FL; D, H, L, P). The basement membrane of the developing urothelium has been outlined in black as a point of reference in 10X and 20X FL views.

**Figure 11:**
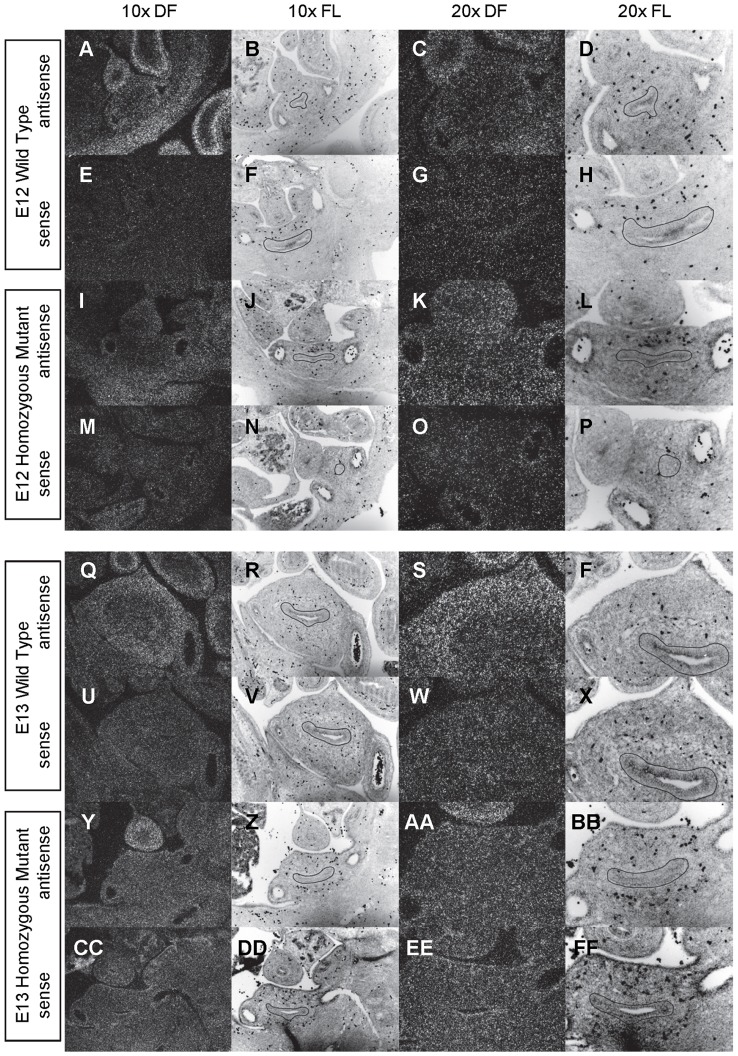
Expression of *Gli3* in the bladder of E12 and E13 mice. *In situ* hybridization analysis of *Gli3* expression in E12 (A-P) and E13 (Q-FF) bladders using antisense (A-D, I-L, Q-T, Y-BB) and sense (E-H, M-P, U-X, CC-FF) riboprobes on transverse sections of wild type (A-H, Q-X) and *mgb−/−* (I-P, Y-FF) mice. Sections are shown at 10X dark field (DF; A, E, I, M, Q, U, Y, CC), 10X fluorescence (FL; B, F, J, N, R, V, Z, DD), 20X dark field (DF; C, G, K, O, S, W, AA, EE) and 20X fluorescence (FL; D, H, L, P, T, X, BB, FF). The basement membrane of the developing urothelium has been outlined in black as a point of reference in 10X and 20X FL views.

**Figure 12:**
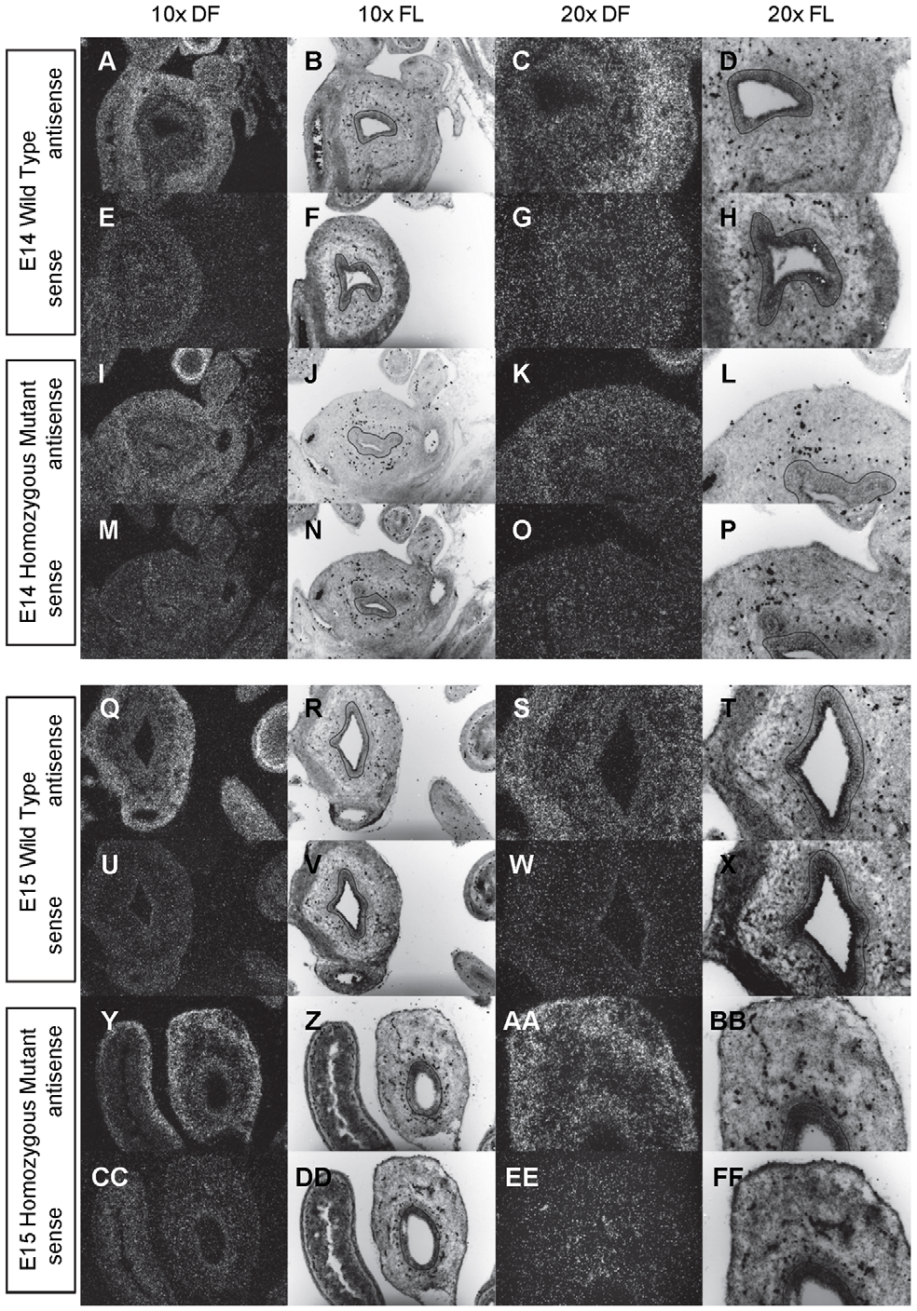
Expression of *Gli3* in the bladder of E14 and E15 mice. *In situ* hybridization analysis of *Gli3* expression in E14 (A-P) and E15 (Q-FF) bladders using antisense (A-D, I-L, Q-T, Y-BB) and sense (E-H, M-P, U-X, CC-FF) riboprobes on transverse sections of wild type (A-H, Q-X) and *mgb−/−* (I-P, Y-FF) mice. Sections are shown at 10X dark field (DF; A, E, I, M, Q, U, Y, CC), 10X fluorescence (FL; B, F, J, N, R, V, Z, DD), 20X dark field (DF; C, G, K, O, S, W, AA, EE) and 20X fluorescence (FL; D, H, L, P, T, X, BB, FF). The basement membrane of the developing urothelium has been outlined in black as a point of reference in 10X and 20X FL views.

**Figure 13:**
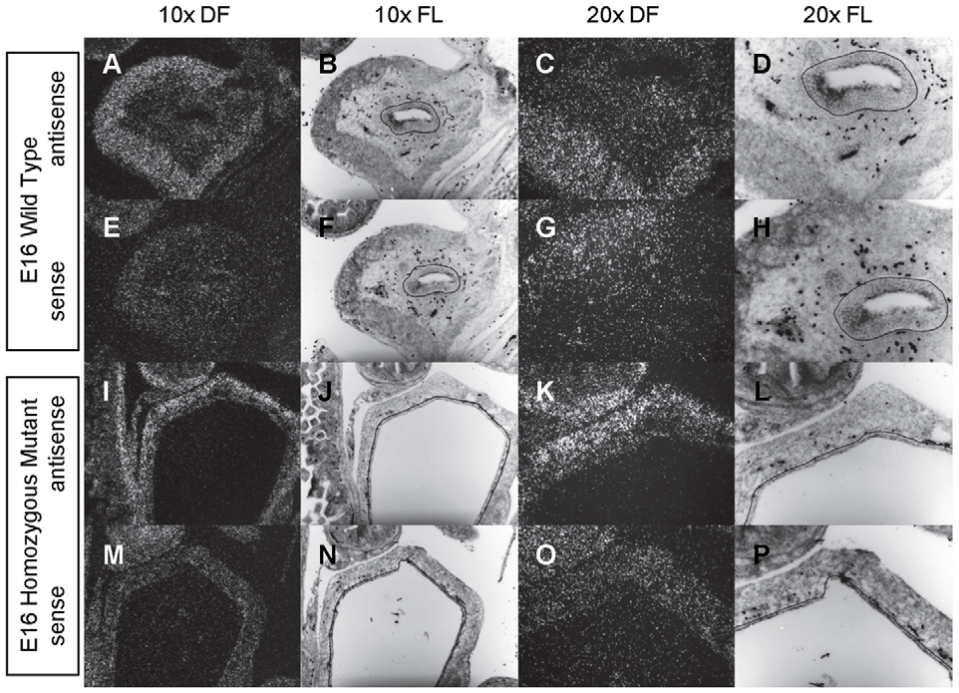
Expression of *Gli3* in the bladder of E16 mice. *In situ* hybridization analysis of *Gli3* expression in E16 (A-P) bladders using antisense (A-D, I-L) and sense (E-H, M-P) riboprobes on transverse sections of wild type (A-H) and *mgb−/−* (I-P) mice. Sections are shown at 10X dark field (DF; A, E, I, M), 10X fluorescence (FL; B, F, J, N), 20X dark field (DF; C, G, K, O) and 20X fluorescence (FL; D, H, L, P). The basement membrane of the developing urothelium has been outlined in black as a point of reference in 10X and 20X FL views.

**Figure 14:**
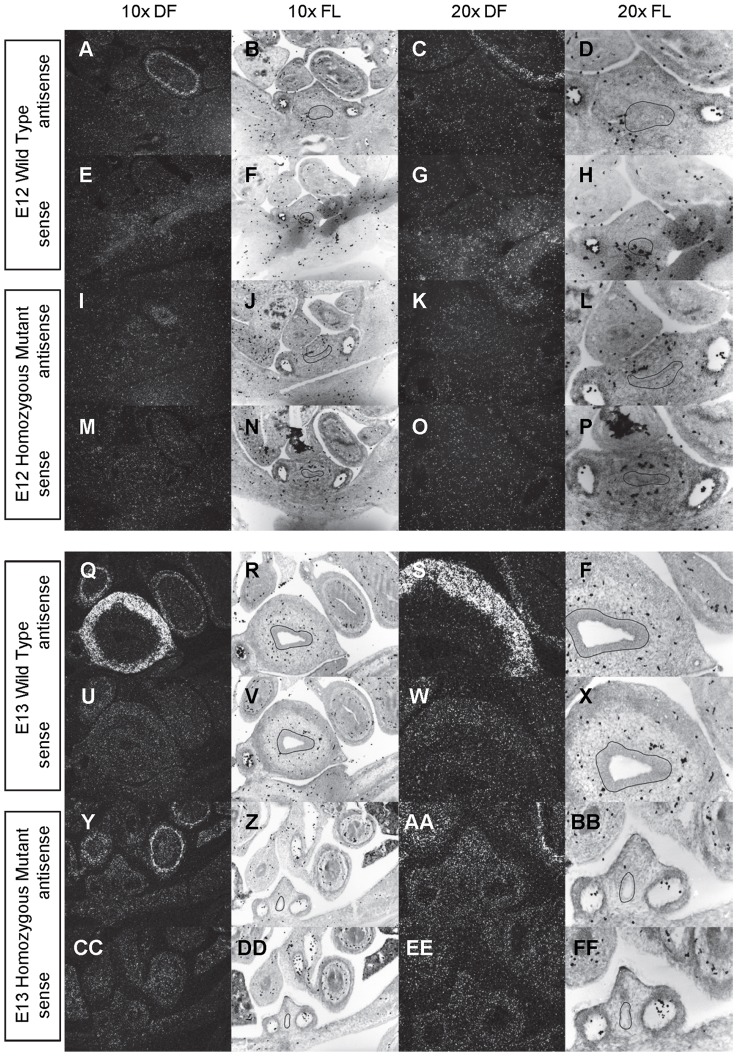
Expression of *Myocd* in the bladder of E12 and E13 mice. *In situ* hybridization analysis of *Myocd* expression in E12 (A-P) and E13 (Q-FF) bladders using antisense (A-D, I-L, Q-T, Y-BB) and sense (E-H, M-P, U-X, CC-FF) riboprobes on transverse sections of wild type (A-H, Q-X) and *mgb−/−* (I-P, Y-FF) mice. Sections are shown at 10X dark field (DF; A, E, I, M, Q, U, Y, CC), 10X fluorescence (FL; B, F, J, N, R, V, Z, DD), 20X dark field (DF; C, G, K, O, S, W, AA, EE) and 20X fluorescence (FL; D, H, L, P, T, X, BB, FF). The basement membrane of the developing urothelium has been outlined in black as a point of reference in 10X and 20X FL views.

**Figure 15:**
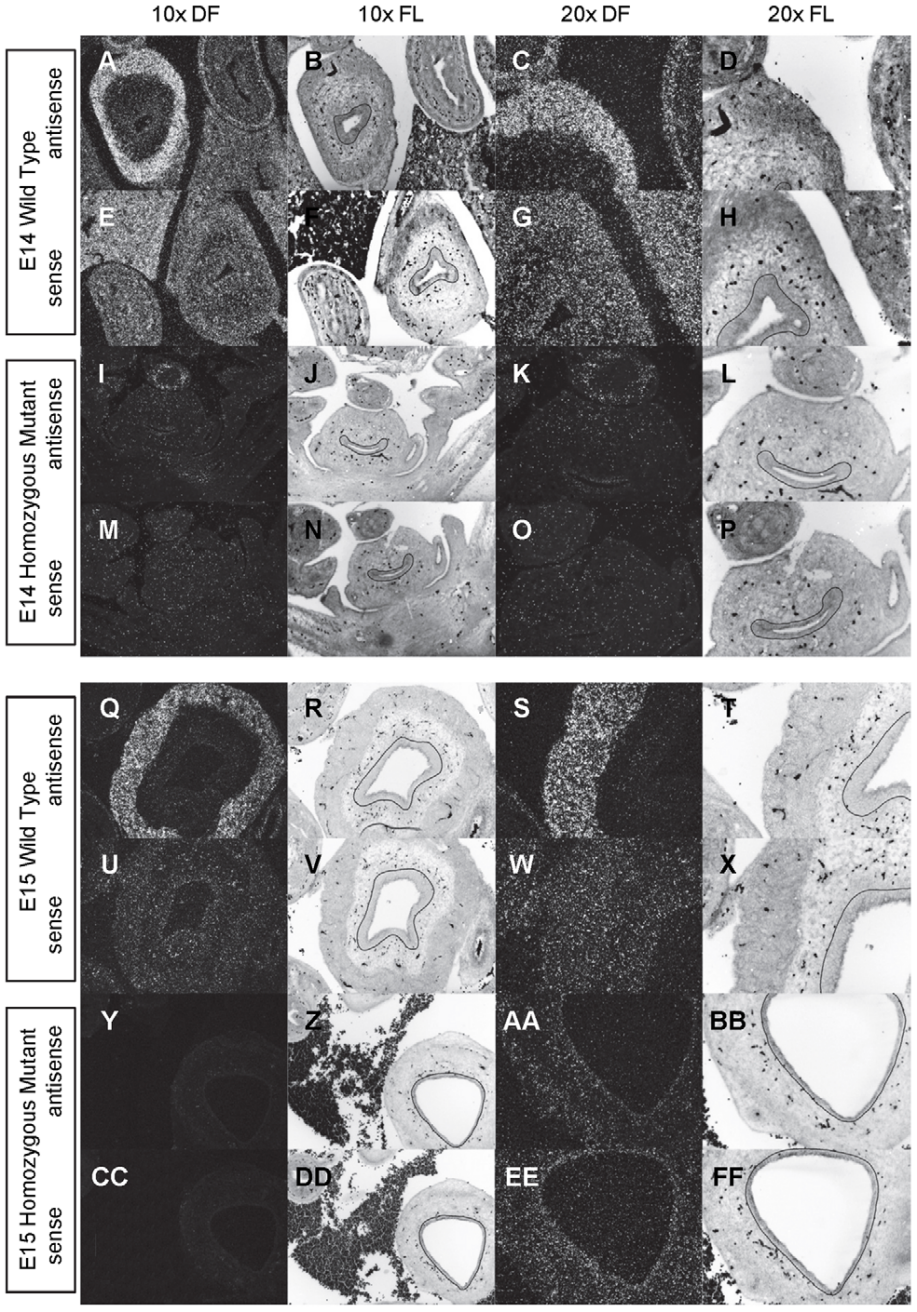
Expression of *Myocd* in the bladder of E14 and E15 mice. *In situ* hybridization analysis of *Myocd* expression in E14 (A-P) and E15 (Q-FF) bladders using antisense (A-D, I-L, Q-T, Y-BB) and sense (E-H, M-P, U-X, CC-FF) riboprobes on transverse sections of wild type (A-H, Q-X) and *mgb−/−* (I-P, Y-FF) mice. Sections are shown at 10X dark field (DF; A, E, I, M, Q, U, Y, CC), 10X fluorescence (FL; B, F, J, N, R, V, Z, DD), 20X dark field (DF; C, G, K, O, S, W, AA, EE) and 20X fluorescence (FL; D, H, L, P, T, X, BB, FF). The basement membrane of the developing urothelium has been outlined in black as a point of reference in 10X and 20X FL views.

**Figure 16:**
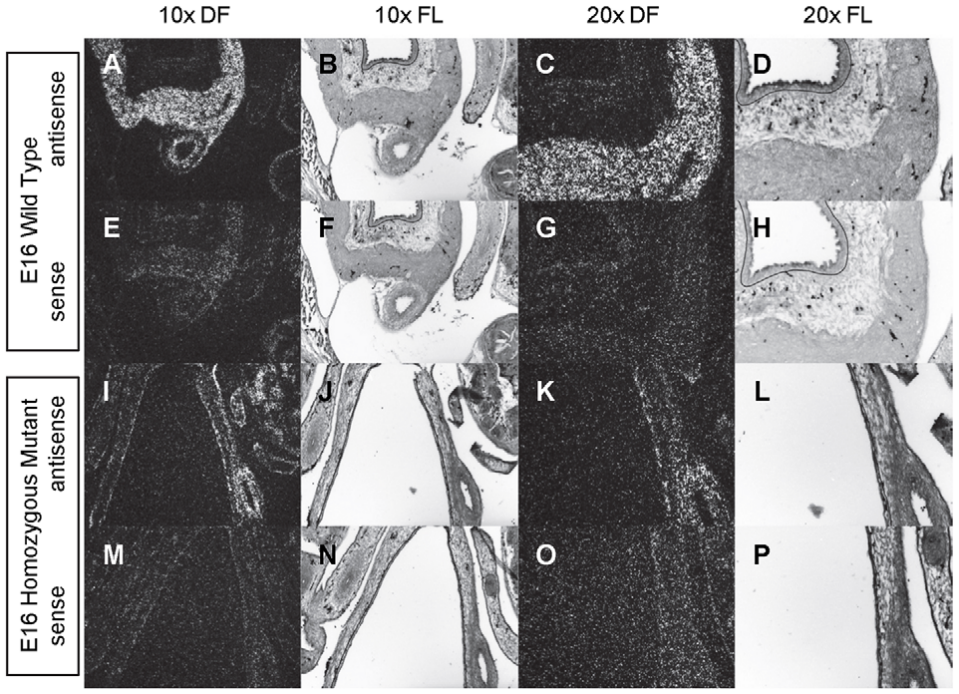
Expression of *Myocd* in the bladder of E16 mice. *In situ* hybridization analysis of *Myocd* expression in E16 (A-P) bladders using antisense (A-D, I-L) and sense (E-H, M-P) riboprobes on transverse sections of wild type (A-H) and *mgb−/−* (I-P) mice. Sections are shown at 10X dark field (DF; A, E, I, M), 10X fluorescence (FL; B, F, J, N), 20X dark field (DF; C, G, K, O) and 20X fluorescence (FL; D, H, L, P). The basement membrane of the developing urothelium has been outlined in black as a point of reference in 10X and 20X FL views.

**Figure 17:**
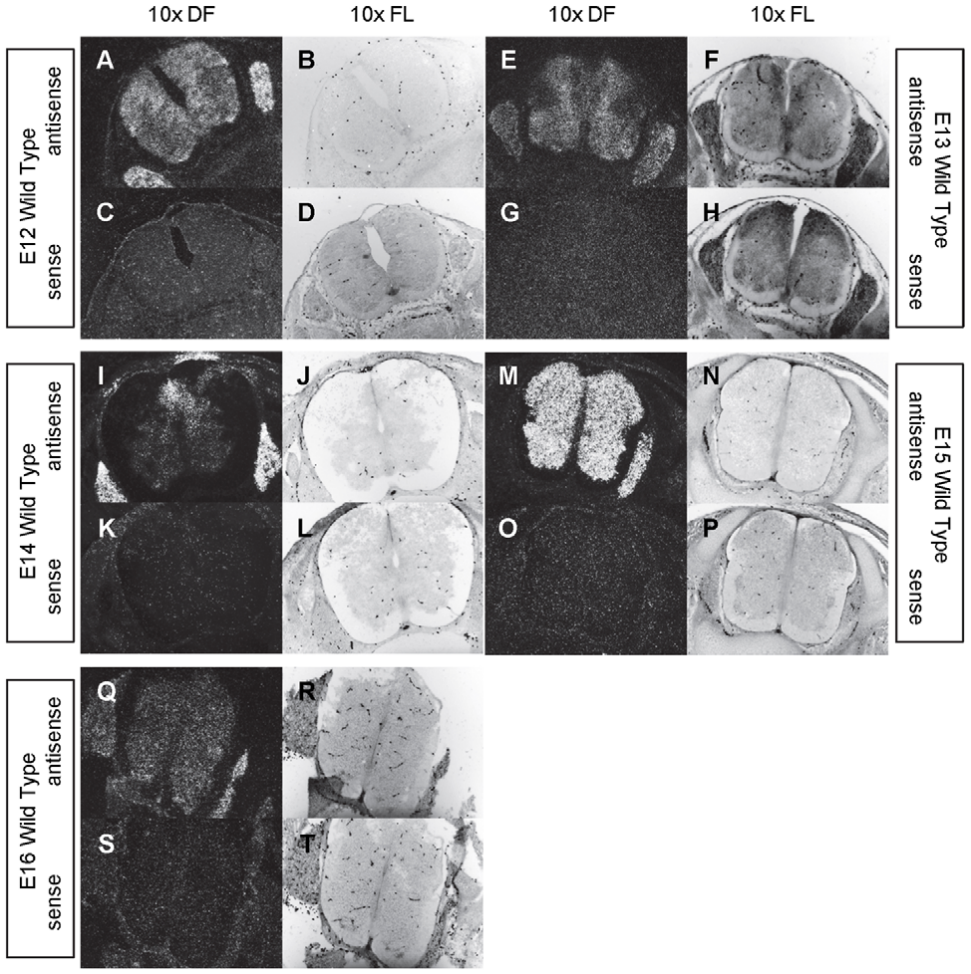
Expression of *β-tubulin* in the central nervous system of E12 through E16 mice. *In situ* hybridization analysis of *β-tubulin* expression in E12 (A-D), E13 (E-H), E14 (I-L), E15 (M-P), and E16 (Q-T) central nervous system using antisense (A-B, E-F, I-J, M-N, Q-R) and sense (E-D, G-H, K-L, O-P, S–T) riboprobes on transverse sections of wild type (A-T) mice. Sections are shown at 10X dark field (DF; A, C, E, G, I, K, M, O, Q, S) and 10X fluorescence (FL; B, D, F, H, J, L, N, P, R, T).

No gender-specific differences in expression of *Shh, Ptch, Gli1, Gli2, Gli3*, or *Myocd* were observed at any of the developmental time points examined in this study. The expression of *Shh, Ptch, Gli1, Gli2, Gli3*, and *Myocd* were examined by antisense and sense RNA probes at each developmental time point assessed. Representative images for each of these are provided to evaluate signal vs. background (Fig. 2–16) and these results are summarized in Table 1. A positive control probe, β-tubulin (*β-tub*), was examined by antisense and sense RNA probes. *β-tub* expressed in multiple tissues, including the central nervous system, in all the developmental time points examined in this study (Fig. 17).

**Table 1:** Summary of *Shh, Ptch, Gli1, Gli3,* and *Myocd* mRNA Expression in the Developing Bladder of WT and *mgb−/−* Mice.

Probe	Stage	WT Urothelium	WT Inner Mesenchyme	WT Outer Mesenchyme	*mgb−/−* Urothelium	*mgb−/−* Inner Mesenchyme	*mgb−/−* Outer Mesenchyme
Shh	E12	++	−	−	++	−	−
	E13	++	−	−	++	−	−
	E14	++	−	−	++	−	−
	E15	++	−	−	++	−	−
	E16	++	−	−	++	−	−
Ptch	E12	−	+	+	−	+	+
	E13	−	+	−/+	−	−/+	−
	E14	−	+++	−	−	++	−
	E15	−	+++	−	−	++	−
	E16	−	+++	−	−	++	−
Gli1	E12	−	−/+	−/+	−	−/+	−/+
	E13	−	++	++	−	+	−/+
	E14	−	++	+	−	++	++
	E15	−	++	++	−	++	+
	E16	−	++	−	−	++	++
Gli3	E12	−	−	−	−	−	−
	E13	−	−	++	−	−	−
	E14	−	−	++	−	−	+
	E15	−	−	++	−	−	++
	E16	−	−	++	−	−	++
Myocd	E12	−	−	−	−	−	−
	E13	−	−	+++	−	−	−
	E14	−	−	+++	−	−	−/+
	E15	−	−	+++	−	−	−
	E16	−	−	+++	−	−	−/+

*Shh*, *Ptch*, *Gli1*, *Gli3*, and *Myocd* mRNA expression in the developing bladder of wild type (WT) and megabladder (*mgb−/−*) mice at embryonic day (E) 12, 13, 14, 15, and 16. Relative expression is reported for mRNA as: (−) no expression, (−/+) faint, (+) low, (++) moderate, and (+++) and high levels of expression. The relative levels of mRNA expression reported for each time-point represent an average of multiple sections processed in a minimum of three separate runs.

### Sonic Hedgehog is expressed in bladder urothelium

*Shh* is uniformly expressed at moderate to high levels in the bladder urothelium of normal mice from E12 to E16 (Fig. 2–4). An identical pattern of urothelium-specific expression of *Shh* is also observed in the developing bladders of *mgb−/−* mice (Fig. 2–4). The development of megabladder by E16 results in significant attenuation of the bladder wall and subsequent pattern of *Shh* expression within the urothelium. A similar epithelial-specific expression of *Shh* is also noted in other visceral organs including the stomach, intestine, colon and rectum with no differences observed between normal and *mgb−/−* mice (Fig. 2–4).

### Patched is expressed in bladder inner mesenchyme

At E12, *Ptch* is expressed at moderate levels throughout the entire bladder mesenchyme from the base of the urothelium to the outer serosa (Fig. 5). By E13, *Ptch* expression begins to restrict towards the inner mesenchyme resulting in low levels of *Ptch* expression throughout the bladder mesenchyme, with an intense band of *Ptch* expression within the inner mesenchyme underlying the urothelium (Fig. 5). As bladder development progresses from E14 to E16, *Ptch* expression becomes sequentially more restricted to the base of the bladder urothelium resulting in little or no expression of *Ptch* in the outer mesenchyme/detrusor smooth muscle and outer half of the inner mesenchyme (Fig. 6–7).

At E12, *Ptch* is expressed at moderate levels throughout the entire developing mesenchyme of *mgb−/−* bladders (Fig. 5). By E13, *Ptch* expression begins to restrict to the inner mesenchyme of *mgb−/−* bladders even though the overall intensity of *Ptch* expression appears reduced versus normal bladders (Fig. 5). At E14, the restriction of *Ptch* expression to the inner mesenchyme of *mgb−/−* bladders appears delayed, resembling the pattern observed in E13 normal bladders (Fig. 5 vs. 6). The further restriction and loss of *Ptch* expression in the distal mesenchyme of E15 *mgb−/−* bladders remains delayed and appears reduced in intensity versus normal bladders (Fig. 6). The highly attenuated E16 megabladder shows intense *Ptch* expression in the lamina propria underlying the urothelium similar to normal bladders although, unlike normal bladders, variable regions of diffuse *Ptch* expression are still observed within the outer mesenchyme (Fig. 7).

*Ptch* expression is also observed in the developing mesenchyme of the stomach, intestine, colon and rectum in both normal and *mgb−/−* mice with no differences in genotype observed (Fig. 5–7). The overall intensity and spatial redistribution of *Ptch* expression during the development of these visceral tissues appears similar to that observed in the bladder with variable maintenance of diffuse *Ptch* expression throughout the inner and/or outer mesenchyme.

### Gli1 is expressed in bladder inner mesenchyme

At E12, *Gli1* is diffusely expressed throughout the entire mesenchyme of normal bladders (Fig. 8). By E13, *Gli1* is moderately expressed in three concentric rings within the bladder mesenchyme that morphologically correspond to an intense ring of expression immediately underlying the urothelium, a light ring of expression in the remaining inner mesenchyme, and an intense ring of expression in the outer mesenchyme (Fig. 8). As bladder development progresses from E14 to E16, *Gli1* expression becomes progressively restricted to the base of the urothelium such that by E16 modest *Gli1* expression is principally observed in the inner mesenchyme underlying the bladder urothelium with no more than light and spotty *Gli1* expression observed in the outer mesenchyme/detrusor smooth muscle (Fig. 9–10).

At E12, *Gli1* is expressed at low levels throughout the developing mesenchyme of *mgb−/−* bladders in a manner similar to normal bladders (Fig. 8). At E13, mesenchymal expression of *Gli1* in *mgb−/−* bladders appears reduced and less organized than that observed in normal bladders (Fig. 8). The development of differentially expressed concentric rings of *Gli1* within the bladder mesenchyme is absent in E14 *mgb−/−* bladders, with the overall intensity and pattern of *Gli1* expression more reminiscent of E13 normal bladders (Fig. 9C vs. Fig. 9K). This less organized pattern of expression within the *mgb−/−* bladder remains at E15, with a slight decrease in *Gli1* expression observed throughout the mesenchyme except in the region underlying the urothelium (Fig. 9). The highly attenuated E16 *mgb−/−* megabladder continues to express *Gli1* within the mesenchymal compartment but, unlike normal bladders, expression is not restricted to the inner mesenchyme at the base of the urothelium. Rather, *Gli1* is expressed throughout the entire E16 bladder mesenchyme (Fig. 10).

A similar mesenchyme-specific pattern of *Gli1* expression is also observed in the developing stomach, intestine, colon and rectum in both normal and *mgb−/−* mice with no differences in genotype observed (Fig. 8–10). The overall intensity and spatial redistribution of *Gli1* expression during the development of these visceral tissues appears similar to that observed in the bladder, with moderate intensity expression throughout the mesenchyme at early development, which was restricted to the inner mesenchyme as development progressed.

### Gli2 expression undetectable

Specific *Gli2* expression was not detected in any of the bladder sections examined in this study even though two different *Gli2* probes were tested including one developed in the Joyner lab [19] and one purchased from Open BioSystems (EMM1002-2414207) (data not shown). This observation, coupled with the success of the remaining *in situ* probes used in this study, suggests that these negative results are not due to technical issues, though it still remains plausible we were unable to detect *Gli2* expression in the developing bladder due to probe specificity problems and/or experimental conditions.

### Gli3 is expressed in bladder outer mesenchyme

At E12, *Gli3* is undetectable in the bladder mesenchyme (Fig. 11). By E13, faint *Gli3* expression is visible in the outer mesenchyme of the bladder (Fig. 11). As bladder development progresses, *Gli3* expression increases moderately within the outer mesenchyme of E14 bladders and is maintained at this level through E16 (Fig. 12–13).

*Gli3* is expressed in the outer mesenchyme of *mgb−/−* bladders similar to normal bladders however; its expression appears delayed, not being detected until E14 (Fig. 11–12). By E15, *Gli3* is expressed at moderate levels within the outer mesenchyme similar to the pattern observed in E15 normal bladders, although the overall expression appears more irregular than normal bladders (Fig. 12S vs. Fig. 12AA). This moderate level of *Gli3* expression within the outer mesenchyme is maintained in the highly attenuated E16 *mgb−/−* bladder (Fig. 13).

A similar mesenchyme-specific pattern of *Gli3* expression is also observed in the developing stomach, intestine, colon and rectum in both normal and *mgb−/−* mice (Fig. 11–13). The overall intensity and spatial localization of *Gli3* expression during the development of these visceral tissues appears similar to that observed in the bladder, with faint expression in the outer mesenchyme at early development and moderate expression in the outer mesenchyme at later developmental time-points.

### Myocardin is expressed in bladder outer mesenchyme in normal mice but absent in *mgb−/−* mice

*Myocd* expression is not detected in E12 normal bladders (Fig. 14). A high level of *Myocd* expression is detected at E13 in the outer mesenchyme of the developing bladder and continues through E16 when the outer mesenchyme differentiates into detrusor smooth muscle (Fig. 14–16). In contrast, *Myocd* shows minimal to no expression in the developing bladders of E12 to E16 *mgb−/−* mice (Fig. 14–16). A mesenchyme-specific pattern of *Myocd* expression is also observed in the developing stomach, intestine, colon, and rectum in both normal and *mgb−/−* mice (Fig. 14–16). The overall intensity and spatial pattern of *Myocd* expression during the development of these visceral organs appears tissue specific.

### PTCH1-LACZ expression provides a functional readout of the Shh pathway and recapitulates the mRNA pattern of expression

**Figure 18:**
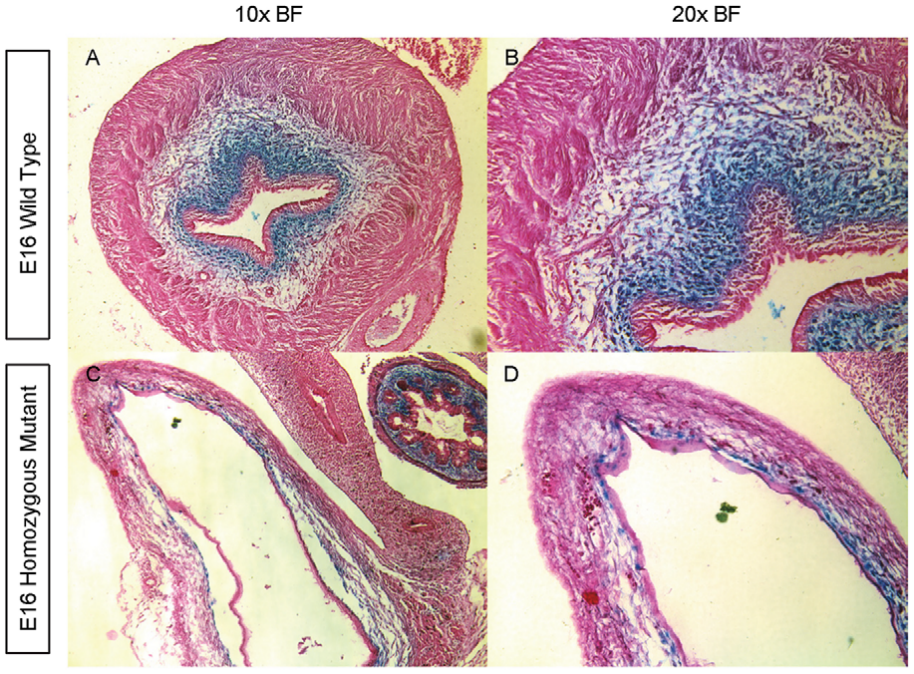
*Ptch* protein and mRNA expression co-localize in E16 bladders. X-gal staining of PTCH-LACZ protein (blue) on transverse sections of E16 bladders in wild type (A, B) and *mgb−/−* (C, D) mice at 10X (A, C) and 20X (B, D) light field.

We examined a functional readout of PTCH expression within the developing bladder by cross breeding normal and *mgb−/−* mice with *Ptch1-LacZ/+* mice [20]. Intense X-gal staining of PTCH is observed in the inner mesenchyme immediately underlying the urothelium of E16 normal bladders, with little to no staining in the outer half of the inner mesenchyme and no staining in the detrusor smooth muscle/outer mesenchyme (Fig. 18). This pattern is identical to the *Ptch* mRNA expression pattern observed in E16 normal bladders using *in situ* hybridization (Fig. 18 vs. Fig. 7).

X-gal staining of PTCH is also present in the inner mesenchyme immediately underlying the urothelium in highly attenuated E16 *mgb−/−* bladders although the overall pattern appears more intermittent than normal bladders. Little to no X-gal staining of PTCH is observed in the remaining mesenchymal compartment of E16 *mgb−/−* bladders. This pattern of X-gal staining of PTCH appears identical to the *Ptch* mRNA expression pattern observed above in E16 *mgb−/−* bladders (Fig. 18 vs. Fig. 7). X-gal staining of PTCH is also observed in developing mesenchyme of the stomach, intestine, colon, and rectum in both normal and *mgb−/−* mice with no differences in genotype observed (Fig. 18).

## Discussion

Normal bladder development requires the precise temporospatial distribution of key morphogenetic signals to generate radial patterning for the subsequent compartmentalization of distinct cellular phenotypes. Radial patterning in the developing bladder initially involves the subdivision of undifferentiated mesenchyme into an inner and outer compartment, which in turn differentiate into the lamina propria and detrusor smooth muscle, respectively (Fig. 1). Although the Shh signaling pathway has been suggested to play a key role in bladder smooth muscle differentiation [14], [16], its precise role in overall bladder patterning and organogenesis remains to be determined. *Shh* is a secreted protein whose canonical activity is mediated by binding to the *Ptch* receptor, de-repression of Smoothened (*Smo*) and subsequent modulation of the Gli-family of transcription factors. SHH patterns tissue development in PTCH expressing cells based upon both their position within the SHH gradient along with the differential expression and function of distinct Gli-family members.

Previous studies have examined the Shh signaling pathway in the urogenital sinus and early bladder development where it plays a key role in establishing the cellular populations needed for later bladder morphogenesis [11], [14]. Haraguchi et al. characterized the expression of *Shh, Ptch* and *Gli1* in early urogenital development in the mouse (E10.5–13.5). These studies indicated that peri-cloacal mesenchyme is the precursor to multiple urogenital structures, and that *Shh* mutant mice display hyoplastic external genitalia, pelvic urethra and bladder development. Additional studies by Cheng et al. characterized the expression of *Shh, Ptch, Gli1, Gli2* and *Gli3* in the mouse urogenital sinus at a single developmental time point (E12.5) and examined the functional role of *Shh* signaling in cultured fetal mesenchymal cells. Their results indicate that *Shh, Gli2* and *Bmp4* differentially regulate mesenchymal cell proliferation and differentiation *in vitro* and in *Gli2−/−* mice. These studies indicate that SHH signaling is critical in early urogenital development but provide little evidence regarding the precise role that this signaling pathway plays in later bladder organogenesis. Therefore, we examine the expression patterns of *Shh, Ptch, Gli1, Gli2, Gli3* and *Myocd* at sequential developmental time points in the developing bladder ranging from urogenital sinus formation through late bladder organogenesis. These studies were performed using both normal and *mgb−/−* mice, which are known to contain a previously characterized short-axis bladder mutation that results in detrusor smooth muscle agenesis [18], [21].

*Shh* is expressed at high levels in the endodermally derived urothelium throughout normal bladder development providing a continuous source of diffusible morphogen in a gradient that is highest immediately adjacent to the urothelium. At the earliest stages of bladder development, *Ptch* and *Gli1* are expressed at low levels throughout the entire undifferentiated mesenchyme suggesting a straightforward readout of the radial SHH diffusion gradient within these cells. As bladder development progresses, the rapid restriction of high levels of *Ptch* expression by E13 to the region immediately underlying the urothelium provides a potential molecular sink that could blunt the further spread of SHH within the mesenchymal compartment. A similar pattern of expression and functional role for *Ptch* has been proposed in previous studies [14], [22]. As a consequence, the concentration gradient of SHH within the developing bladder would be significantly altered after E13, creating an inner mesenchymal compartment with high SHH and PTCH expression that differentiates into lamina propria consisting of loose connective tissue and an outer mesenchymal compartment with little or no SHH and PTCH expression that compacts and differentiates into detrusor smooth muscle. These observations are consistent with prior studies indicating that high levels of SHH/PTCH inhibit smooth muscle differentiation, while low levels induce smooth muscle differentiation [11], [14].

The Gli-family of transcription factors has been shown to have distinct and overlapping functions with respect to tissue patterning in development and loss of *Gli* expression has been shown to result in urogenital defects [11], [19], [23], [24]. A variety of studies indicate that GLI1 is a transcriptional activator, while GLI2 and GLI3 can function as both transcriptional activators and repressors [19], [23]–[26]. Our results indicate that *Gli1* is expressed throughout the entire bladder mesenchyme during the early stages of development but becomes restricted to the outer mesenchyme and suburothelial inner mesenchyme by E14 (Fig. 8S vs. Fig. 9C). Interestingly, *Gli2* expression in the developing bladder was not detected in our study. This observation is in contrast to Cheng et al., who reported *Gli2* expression in the mesenchyme surrounding the ventral urogenital sinus at E12.5 and suggested that it played a key role in modulating mesenchymal proliferation and differentiation [11]. These differences most likely reflect variations in embryonic staging as well as the fact that their functional studies were performed *in vitro* using cultured fetal bladder mesenchymal cells. We hypothesize that *Gli2* expression occurs in the mesenchyme surrounding the urogenital sinus leading up to E12.5, where it may play a key role in initiating Shh pathway signaling by activation of *Gli1* in a manner similar to that proposed by Dennler and Bai [26], [27]. Finally, our results indicate that *Gli3* expression initiates in and remains restricted to the outer mesenchyme, providing a selective expression pattern that delimits this presumptive smooth muscle region from the rest of the developing bladder as early as E13.

**Figure 19:**
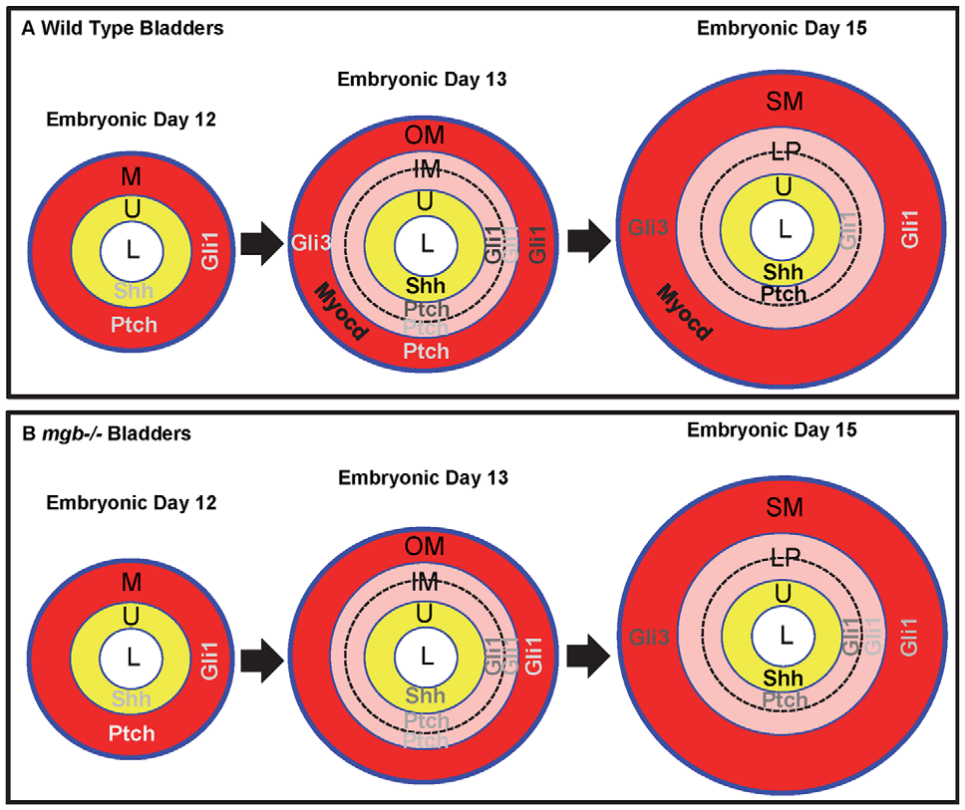
Temporospatial distribution of *Shh, Ptch, Gli1, Gli3*, and *Myocd* mRNA expression during murine bladder development. Differential expression patterns of *Shh, Ptch, Gli1, Gli3*, and *Myocd* mRNA at low (light gray), moderate (medium gray) and high (black) levels in the urothelium (U; yellow), mesenchyme (M, red), inner mesenchyme (IM; pink), outer mesenchyme (OM; red), lamina propria (LP; pink) and detrusor smooth muscle (SM; red) are shown in E12, E13 and E15 normal and *mgb−/−* bladders. Note the temporal and spatial differences in expression in *mgb−/−* bladders suggestive of a developmental delay in short axis patterning that may contribute to an absence of *Myocd* expression and subsequent detrusor smooth muscle development.

These expression patterns indicate that by E14 the developing bladder is subdivided into four distinct regions that including the 1) **↑** *Shh*-positive urothelium, 2) **↑** *Ptch* and **↑** *Gli1*-positive suburothelial inner mesenchyme, 3) non-*Gli* expressing inner mesenchyme and 4) **↓** *Ptch*, **↓** *Gli1* and **↑** *Gli3*-positve outer mesenchyme (Fig. 19). As discussed above, the suburothelial inner mesenchyme that is expressing high levels of *Ptch* and *Gli1* may act as a molecular sink as bladder development progresses, thereby dynamically altering the SHH radial gradient to promote further differentiation of the bladder mesenchyme. The high levels of SHH detected by the *Ptch* and previously reported *Gli2* expressing cells of the inner mesenchyme would be predicted to support cellular proliferation and suppress smooth muscle differentiation within the presumptive lamina propria [11]. In contrast, the *Gli3*-positive outer mesenchymal cells that also express low levels of *Ptch* and *Gli1* would be exposed to low levels of SHH. Li et al. states that *Gli3* functions as an activator in the presence of SHH but acts as a repressor in its absence due to proteolytic cleavage of the C-terminus [23]. Acting as a repressor in the outer mesenchyme of the bladder, *Gli3* may block high levels of cell proliferation thereby supporting the subsequent differentiation of detrusor smooth muscle.

*Myocd* is a SAP-family transcriptional co-factor that plays a key role in the development of both cardiac and smooth muscle [28], [29]. At least two distinct *Myocd* isotypes have been described including a full-length cardiac-*Myocd* (*cMyocd*) and truncated smooth muscle-*Myocd* (*sMyocd*). *sMyocd* interacts with serum response factor (*SRF*) to form a transcriptional complex that activates smooth muscle-specific gene expression [28]–[30]. High levels of *sMyocd* expression were observed in the outer mesenchyme of the developing bladder from E13 through E16 (Fig. 14–16). The high levels of *sMyocd* expression occur in concordance with low levels of *Gli3* expression within the outer mesenchyme, but appear to precede the more obvious radial compartmentalization demarcated by *Ptch* and *Gli1* expression at later developmental time points. These observations suggest that *sMyocd* may not be a direct downstream transcriptional target of the Shh signaling pathway but rather is expressed independently during early bladder development, thereby priming the outer mesenchyme for smooth muscle differentiation based upon subsequent radial patterning cues.

Since the *mgb−/−* mouse possesses a tissue-specific defect in detrusor smooth muscle development [18], [21], [31] and prior studies indicate that the Shh signaling cascade plays a key role in radial patterning and visceral smooth muscle development [10], [28], we examined the Shh signaling pathway in *mgb−/−* bladders. *Mgb−/−* mice expressed normal levels of *Shh* within the bladder urothelium suggesting that this key morphogen is in the right place at the right time and at correct levels necessary to direct normal radial patterning and smooth muscle development in these animals. This observation is consistent with prior studies indicating that urothelial development in *mgb−/−* bladders appears normal [18]. In contrast, the altered levels and pattern of *Ptch* expression observed within *mgb−/−* bladder mesenchyme could potentially have a significant effect on radial patterning in these animals. First, the more diffuse and less intense *Ptch* expression observed within the suburothelial inner mesenchyme of developing *mgb−/−* bladders would be predicted to generate a less efficient molecular sink for SHH binding. Compounding this potential change in the SHH gradient is the lack of detectable levels of *Ptch* expression within the outer mesenchyme of *mgb−/−* bladders versus normal bladders (Fig. 6C vs. Fig. 6K). In addition, *mgb−/−* bladders also showed a 24-hour developmental delay in the expression of both *Gli1* and *Gli3*. Although delayed, *Gli3* expression in *mgb−/−* bladders initiated and remained restricted to the outer mesenchyme similar to the pattern observed in normal bladders (Fig. 11S vs. Fig. 12K). In contrast, *Gli1* expression in *mgb−/−* bladders is never selectively restricted to the suburothelial inner mesenchyme and outer mesenchyme as observed in normal bladders (Fig. 9). Finally, *sMyocd* is not expressed within the outer mesenchyme of *mgb−/−* bladders (Fig. 14–16).

These results indicate that the outer mesenchyme of *mgb−/−* bladders possess a series of overlapping developmental defects that include 1) potentially higher levels of SHH ligand and lower levels of PTCH receptor, 2) temporal and spatial changes in *Gli1* and *Gli3* expression, and 3) a complete absence of *sMyocd* expression (Fig. 19). Although the precise ordering and potential interplay of these defects remains to be determined, it is clear that any one of these changes would be predicted to have profound effects on both the SHH gradient as well as the downstream transcriptional readout of that gradient. The fact that the outer bladder mesenchyme is the precise morphological region that differentiates into detrusor smooth muscle suggests that the combination of these defects within this domain may contribute to the development of an amuscular bladder in *mgb−/−* mice.

At present, it remains unknown whether *Myocd* is a direct transcriptional target of the Shh signaling pathway during bladder smooth muscle development. As discussed above, the earliest demarcation of radial patterning observed in the bladder occurs around E13 when low levels of *Gli3* and high levels of *sMyocd* expression are detected within the outer mesenchyme. If *sMyocd* is transcriptionally activated independent of the Shh signaling pathway at E13, the presence of *Gli3* within the outer mesenchyme may provide a SHH-based repression of cellular proliferation within this same region. This decrease in cellular proliferation would be predicted in turn to down-regulate SRF expression to the low levels necessary for SRF-MYOCD complex formation and subsequent transcriptional initiation of the smooth muscle differentiation program [32], [33]. In this manner, *Shh* signaling pathway could be directly responsible for establishing the overall radial patterning within the bladder, while indirectly supporting the progression of detrusor smooth muscle development independent of direct transcriptional activation of *Myocd* expression.

It is also plausible that the independent expression of both SHH and PTCH in the E12 urogenital sinus could regulate *Myocd* expression through non-canonical *Shh* signaling prior to radial patterning. SHH signaling through PTCH has been shown to drive alterations of cell morphology independent of *Gli*-mediated transcription [34]. Furthermore, SHH can act independent of PTCH through a second transmembrane receptor known as Hip [35]. Alternatively, in the absence of SHH, PTCH1 has been shown to interact with phosphorylated-cyclinB1 to regulate the G_2_/M checkpoint, while PTCH can also function as a positive regulator of SMO to promote cell proliferation [36], [37]. Similar non-canonical Shh signaling pathways may be important during early bladder development where they help to initiate *Myocd* expression within the outer mesenchyme prior to overt morphological signs of radial patterning.

If *Myocd* is not a direct transcriptional target of either canonical or non-canonical *Shh* signaling, then the question remains how might it be regulated? Additional key developmental pathways have been identified as important in smooth muscle differentiation including the Wnt/β-catenin and Fgf signaling pathways [38]–[42]. *Wnt2−/−* null mutants exhibit a loss of *Myocd* expression that, in turn, results in a loss of smooth muscle cells. *Wnt2* appears to activate *Myocd*, *Fgf10* and *Wnt7b*, promoting smooth muscle development by increasing the expression of smooth muscle-specific genes [39]. Interestingly, *Gli3* has been shown to act on *Fgf10* to maintain high expression of smooth muscle-specific genes providing a possible regulatory link between the Wnt/β-catenin and Shh signaling pathways [42].

In summary, this study represents the first comprehensive analysis of the Shh signaling pathway and a potential downstream target (*Myocd*) during key consecutive stages of normal and abnormal bladder development. *Shh, Ptch, Gli1, Gli3* and *Myocd* are all expressed in distinct temporospatial patterns that appear consistent with radial compartmentalization and specialization of the developing bladder. Alterations in both the temporal and spatial distribution of *Ptch, Gli1, Gli3* and *Myocd* were discerned in developing *mgb−/−* bladders in a pattern consistent with the lack of detrusor smooth muscle development detected in these animals. These observations highlight the importance of the Shh signaling pathway in radial bladder development and indicate that the interplay between this key signaling pathway and *Myocd* expression is highly critical for normal detrusor smooth muscle development. In conclusion, the results of this study provide important insights into the molecular mechanisms controlling both normal and abnormal bladder development and a basis for future studies designed to evaluate therapeutic strategies for the prevention and treatment of urinary tract diseases and disorders.

## Materials and Methods

### Ethics Statement

The IACUC board of The Research Institute at Nationwide Children's Hospital approved all of the following animal studies under the Animal Welfare Assurance Number 02105AR.

### Animals

Embryonic day (E) 12, 13, 14, 15, and 16 *FVB/N* wild type mice and *mgb−/−* mice, previously described, were used for *in situ* hybridization studies as described below [18], [43], [44]. Mice were maintained according to the National Institutes of Health Guide for the Care and Use of Laboratory Animals. E12-E16 wild type and *mgb−/−* mice were genotyped by real-time quantitative PCR using specific primers sets as previously reported [18]. Male and female embryos of both genotypes (wild type and *mgb−/−*) at each time-point (E12-E16) were analyzed for every probe evaluated in this study. A minimum of N = 3 (and up to an N = 6) of embryos were assessed for each probe. Timed-pregnancy matings and embryo harvests were performed at the same time of the day to help ensure the embryos assessed for each time-point were at very similar stages of development and accurate representations of the time-point being evaluated.

### PCR and Gender-Determination

DNA was isolated using the Spin Doctor Genomic DNA Isolation Kit, according to the manufacturer's protocol (Gerard Biotech). Primers 5′-TGAAGCTTTTGGCTTTGAG-3′ and 5′-CCGCTCGCAAATTCTTTGG-3′ were used to detect the X- and Y-chromosomes by amplification of a X chromosome band and a Y chromosome band as described previously, 95°C for 5 min, (95°C for 30 s, 57°C for 30 s, and 72°C 60 s) ×30 and 72°C for 10 min (Cunningham 2002). The PCR product was confirmed by DNA gel electrophoresis.

### In Situ Hybridization

Embryos from timed matings were removed from wild type and homozygous *mgb−/−* mice and fixed in 4% paraformaldehyde, then transferred to 70% ethanol. Specimens were processed in the Biomorphology Core using standard procedures (TRINCH). Serial paraffin-embedded transverse sections (10 uM) of the bladder were affixed to slides (2–4 sections/slide). *In situ* hybridization using ^35^S-UTP radiolabeled riboprobes was performed on the paraffin-embedded sections from E12-E16 normal and mutant embryos as described previously [45]. Briefly, slides carrying the sections were prehybridized with hybridization solution for 90 minutes while riboprobes were labeled with ^35^S-UTP. The riboprobes were purified with a NucAway kit (Amersham) and the slides were hybridized with 70,000 DPM counts of riboprobe in hybridization solution. Slides were incubated O/N at 50^o^C in a hybridization oven. Following incubation, the slides were washed in high stringency washes (FSM, STE x2, STE + yeast-tRNA and RNase, STE + BME, FSM x2, 2x SSC, 0.1x SSC) and dehydrated in increasing strength ethanol (30%, 50%, 70%, 85%, 95%, 100%). Slides were air-dried, placed on film, and exposed for 3 days. Following exposure, slides were emulsion coated (Kodak), incubated at 4^o^C for 10–14 days, and developed (Developer, water, Fixer, water) (Kodak). Lastly, slides were cover slipped with permaslip and observed under dark field and fluorescence. Probes used included:

1. *Shh* (courtesy of Herman lab, TRINCH).
2. *Ptch* (courtesy of Herman lab, TRINCH).
3. *Gli1* (courtesy of Joyner lab [19]).
4. *Gli2* (courtesy of Joyner lab [19], Openlab BioSystems (EMM1002-2414207)).
5. *Gli3* (courtesy of A. Joyner [19]).
6. *Myocd* (McHugh lab) (ttcctgtgcacactgctgtaaagtccaagtctttgggtgacagtaagaaccgccacaaaaagcccaaagaccccaaaccaaaggtgaagaagctcaaataccatcagtacatccccccagaccagaaggcagagaagtctcccccacccatggactctgcctatgcccggctgctccagcaacagcagctattcctgcagctacagatcctcagccagcagcagcaacagcagcagcaacagcagcagcagcaacagcagcagcagcagcagcggttcagctaccctgggatgcaccaaacacacctcaaagaaccaaatgaacagatggccagaaatccgaatccttcttcaacaccactgagcaatacccctctatcccctgtcaaaaatagcatttctggacaaactggtgtttcttctctcaaaccaggccccctcccacccaacctggatgatctcaaggtgtcagagttaagacaacagcttcgaatccggggcttgccagtgtcaggcaccaagacagcgctggtggaccggcttcgtcccttccaggattgtgctggcaaccctgtgcccaactttggggacatcacaactgtcacctttcctgtcacgcccaacaccttgcccagttatcagtcctccccgacaggcttctaccactttggcagcacaagcttcagc).
7. β-tubulin (McHugh lab) was used as a positive control. (aggagtgtgagcattgcgactgtcttcagggcttccagctcacccactcgctgggcggtggcacgggctcaggcatgggcacactgctcatcagcaagatccgagaggagtacccggaccgcatcatgaacaccttcagcgtcatgccgtcacccaaggtctcagacaccgtggtggagccctacaacgccacattgtcagtgcaccagctggtagagaacaccgacgagacctactgcatcgacaacgaggccctctatgacatctgcttccgcacgctcaagctgaccacacccacttacggggacctcaaccactt).

The primers utilized for both of these novel probe sequences are underlined and derived from published exon sequences.

### X-gal Staining

Timed matings were performed with *Ptch-lacZ/+; mgb−/+* dames crossed to *Ptch-lacZ/+; mgb−/−* males (*Ptch-lacZ/+* mice courtesy of Herman lab) [20]. At embryonic day (E) 16, embryos were harvested and fixed according to established procedure [46]. Briefly, embryos were fixed in a solution of 0.2% gluteraldehyde, 2% formalin, 5 mM EGTA, 2 mM MgCl2, 100 mM potassium phosphate buffer, pH 7.3, for one hour on a horizontal shaker. Following fixation, embryos were rinsed three times for 30 minutes each on a horizontal shaker in a solution of 0.1% sodium deoxycholate, 0.2% NP40, 2 mM MgCl2, 100 mM potassium phosphate buffer, pH 7.3. Embryos were stained for 60 hours in a solution of 1 mg/mL X-gal, 5 mM potassium ferricyanide, and 5 mM potassium ferrocyanide in the rinse solution on a horizontal shaker. Following staining, embryos were briefly rinsed in 1x PBS and transferred to 70% ethanol. Specimens were processed in the Biomorphology Core using standard procedures (TRINCH). Serial paraffin-embedded transverse sections (4 uM) of the bladder were affixed to slides (4–8 sections/slide). Lastly, slides were deparaffinized, counter stained with eosin, and cover slipped with permaslip. Slides were observed under light field.

### Microscopy

We performed light field, dark field, and fluorescent microscopy using a Zeiss Imaging Microscope equipped with 10x and 20× objectives and a digital camera. We imaged transverse sections of bladder, stomach, intestine, colon, and rectum from normal (wild type) and mutant (*mgb−/−*) mice. For *in situ* hybridization analysis, we scored a minimum of three embryos per stage, from E12-E16, for the expression of silver grains. For PTCH-LACZ analysis, we scored E16 embryos for X-gal staining. Identical camera settings were used for dark field and fluorescent images of each stage embryo. We collected the images using OpenLab Software and edited the images with Adobe Photoshop (equal adjustment of Sharpness, Contrast, Shadow, and Brightness; and fluorescent images were inverted).
